# Supplementary material for: Effects of different types of sensory signals on reaching performance in persons with chronic schizophrenia
Source: PLoS One. 2020 Jun 24;15(6):e0234976. doi: 10.1371/journal.pone.0234976 (PMC7314021; doi:10.1371/journal.pone.0234976)
Supplement: S1 Dataset — (PDF) [file pone.0234976.s001.pdf]

	Reaction Time / Inter-response			
	schizophrenia		healthy	
	mean	std	mean	std
visual	496.57	27.31	395.2	27.31
auditory	544.75	47.88	524.72	47.88
none	610.96	43.52	247.67	43.52

	Movement Time			
	schizophrenia		healthy	
	mean	std	mean	std
visual	355.2	15.77	273.7	15.77
auditory	742.5	32.52	563.5714	32.52
none	380.99	35.53	274.01	35.53

	Peak Velocity			
	schizophrenia		healthy	
	mean	std	mean	std
visual	104.65	7.74	158.15	7.74
auditory	102.79	6.61	152.81	6.61
none	101.89	6.37	156.9471	6.37

	Percentage of Time			
	schizophrenia		healthy	
	mean	std	mean	std
visual	42.88	1.33	44.47	1.33
auditory	46.07	1.43	45.58	1.43
none	47.62	0.82	43.9	0.82

	Movement Unit			
	schizophrenia		healthy	
	mean	std	mean	std
visual	1.71	0.1	1.16	0.1
auditory	1.72	0.08	1.18	0.08
none	1.46	0.08	1.15	0.08
