# Supplementary material for: Effects of different types of sensory signals on reaching performance in persons with chronic schizophrenia
Source: PLoS One. 2020 Jun 24;15(6):e0234976. doi: 10.1371/journal.pone.0234976 (PMC7314021; doi:10.1371/journal.pone.0234976)
Supplement: Attachment — Submitted filename: Detailed Response to Reviewers(PONE-D-19-26482)2.doc [file pone.0234976.s005.doc]

**Detailed Response to Reviewers**

**Manuscript ID number: PONE-D-19-26482**

**Responses to Reviewer #1:**

We appreciate that the reviewer’s comments. The followings are our point-by-point responses. Your comments are quoted in black font, our responses are in blue font and all the changes made in the revised manuscript are marked using the Microsoft Word's Track changes. The page and line numbers are in accordance with the highlighted version.

a few minor issues remain to be resolved:

abstract: final sentence is misleading. Authors did not test whether motor performance was linked to clinical status, except for CPZ and DOI. Please omit this information in the abstract
**[Reply]: the final sentence”…..** independent of clinical characteristics. **“ has been changed to “**… **independent of duration of illness and antipsychotic dosage.**”

table 1 Levels of antipsychotics should be "dosage of antipsychotics" because authors do not report blood levels
**[Reply]: corrected as suggested, Line 239 Table 1**

results: again please delete the phrase on clinical characteristics (correlation with motor), but instead state that DOI and CPZ did not correlate (line 265 ff)
**[Reply]: Line, 256, the sentence has been changed to “The results of Pearson’s correlations showed that reaching performance neither significantly correlated with duration of illness nor with antipsychotic dosage. “**

discussion and throughout manuscript: please replace "schizophrenic patients" with "schizophrenia patients" or "patients with schizophrenia". Schizophrenic is considered to be a stigmatizing adjective
**[Reply]: corrected as suggested**

**Detailed Response to Reviewers**

**Manuscript ID number: PONE-D-19-26482**

**Responses to Reviewer #2:**

We appreciate that the reviewer’s comments. The followings are our point-by-point responses. Your comments are quoted in black font, our responses are in blue font and all the changes made in the revised manuscript are marked using the Microsoft Word's Track changes. The page and line numbers are in accordance with the highlighted version.

Reviewer #2: In this revised manuscript the authors have taken into consideration some of my previous comments but they did not address adequately several others and especially my criticism which is presented in comment number 3.
As I pointed out one cannot directly compare the time interval between successive movements in a self-paced sequence movement task which is the no signal condition with the externally triggered visual and auditory task in which the time of onset for each response is the reaction time. The authors just renamed RT in the no signal task to IRI. **What they should have done is a separate analysis of RT in the visual and auditory conditions comparing patients and controls and a separate analysis for IRI in the self-paced movement task comparing the IRI between patients and controls.** The discussion of these two measures should also be separate because different cognitive processes are engaged in self-paced sequential movements and different processes are engaged in externally driven RT processes. The authors do mention the relevant neural systems that are engaged in self-paced sequential movements that involve the prefrontal cortex and particularly area SMA. Indeed sequential movements are pre-programmed and executed in a unified manner. The authors observe a large difficulty of patients in initiating each component of the sequence of these sequential movements and this is very relevant to the difficulty of the patients in maintaining the sequence in working memory and initiating each movement in the sequence. This effect is different from the difficulty of the patients in initiating externally triggered movements to a target where the system involves the allocation of attention to the external stimulus and the programming of a response to this stimulus. A different fronto-parietal network is activated in these stimulus response tasks. In essence the authors observe differences between patients and controls in two different systems, one related to the programming of a sequence of movements and one to attending and responding to external stimuli. This is why I still think the authors should perform separate analyses for RT and IR and discuss the differences between patients and controls separately for RT and IRI.

**[Reply]: You have raised an important point here and suggested that we should separate analysis of RT in the visual and auditory conditions comparing patients and controls and a separate analysis for IRI in the self-paced movement task comparing the IRI between patients and controls. However, we believe that combine the data to analyze would be more appropriate because combined a 2×2 two way ANOVA and one t-test to a 2×3 two way ANOVA allowed us to avoid the problem of multiple comparisons. In this study, the same experimental task (reaching out and return) was performed in three conditions. We don’t think it is inappropriate to directly compare reaching performance in the auditory, visual and self- paced target conditions.**

**In the literature, there were also some studies that compared self-initiated and externally triggered movements directly (Cunnington, et al., 2002, Jenkins, et. al., 2000, Gilbert, et al., 2009).**

Cunnington, R., Windischberger, C., Deecke, L., & Moser, E. (2002). The preparation and execution of self-initiated and externally-triggered movement: a study of event-related fMRI. Neuroimage, 15(2), 373-385. doi:10.1006/nimg.2001.0976

Jenkins, I. H., Jahanshahi, M., Jueptner, M., Passingham, R. E., & Brooks, D. J. (2000). Self-initiated versus externally triggered movements: II. The effect of movement predictability on regional cerebral blood flow. Brain, 123(6), 1216-1228. doi:10.1093/brain/123.6.1216

Gilbert, S. J., Gollwitzer, P. M., Cohen, A. L., Burgess, P. W., & Oettingen, G. (2009). Separable brain systems supporting cued versus self-initiated realization of delayed intentions. J Exp Psychol Learn Mem Cogn, 35(4), 905-915. doi:10.1037/a0015535

**Behavioral data. Left panel: Mean percentage of prospective memory (PM) targets detected in the self-initiated and cued conditions. Right panel:** Mean reaction time (RT) in the ongoing tasks. **Pale gray bars indicate RTs during the ongoing task at the beginning of the experiment, before prospective memory (PM) instructions were introduced. Black bars indicate reaction times after PM instructions were introduced. Error bars indicate standard error of the mean.**

IN response to my comment on the effects of medication the authors report no correlation of medication to all measured variables. Could they provide the r coefficients in a table or a supplementary table?

**[Reply]: Line 265, all the correlation coefficients has been shown in Table 2**

In response to my comment about the measurement of peak velocity and movement units there is still no clear answer. I commented in my review that the subjects perform two arm movements each time one reaching out to the target and one returning back to the home position and pressing the start button. The authors explained that the MT was the time from release of the home button to the pressing of it so MT corresponds to both arm movements. What about PV and MU? The authors say in their response that they get these measurements from the “zebris” system. That was not my question. My question was WHICH movement’s profile was used to measure these parameters? The reaching out movement or the returning movement or both?

**[Reply]: Line 155, the sentence has been changed to “RT/IRI and MT of total movement (reaching out to touch and return) were measured by the Interactive Sound and Light Eye-Hand Coordination Training System, and the other velocity parameters (PV, PPV, and MU) of reaching out to touch were measured by Zebris.**” **for clarity.**

The description of the results need rephrasing, for example the phrase: “The persons with chronic schizophrenia demonstrated much more RT, MT, and MU but less PV compared to healthy subjects when performing the reaching task” is very bad English and should be re-written. The whole manuscript also needs to be carefully edited by a native English speaker.

**[Reply]: Line 246, the sentence has been changed to “ The persons with chronic schizophrenia showed significantly greater RT, MT, and MU but lower PV compared to healthy subjects when performing the reaching task.**”

**I have sent my manuscript to** [**https://www.servicescape.com/editors/acadconsult**](https://www.servicescape.com/editors/acadconsult) **to edit before my first submission and I sent it to edit again before I re-submit the revised manuscript. The background of the editor is as follows, therefore, I supposed that she is a native English speaker and she is qualified to edit the manuscript.**

**AcadConsult	(Gabriela Dye, PhD)**

Credentials

- Ph.D. in Psychology from the University of Rhode Island
- M.A. in Psychology from the University of Rhode Island
- B.S. in Psychology from Old Dominion University

**The editor re-read and edited the entire revised paper again. The paper has been carefully revised to improve the readability.**
